# Supplementary figures and images for: Genome-Wide Analysis of Gene Expression Noise Brought About by Transcriptional Regulation in Pseudomonas aeruginosa
Source: mSystems. 2022 Nov 15;7(6):e00963-22. doi: 10.1128/msystems.00963-22 (PMC9765613; doi:10.1128/msystems.00963-22)

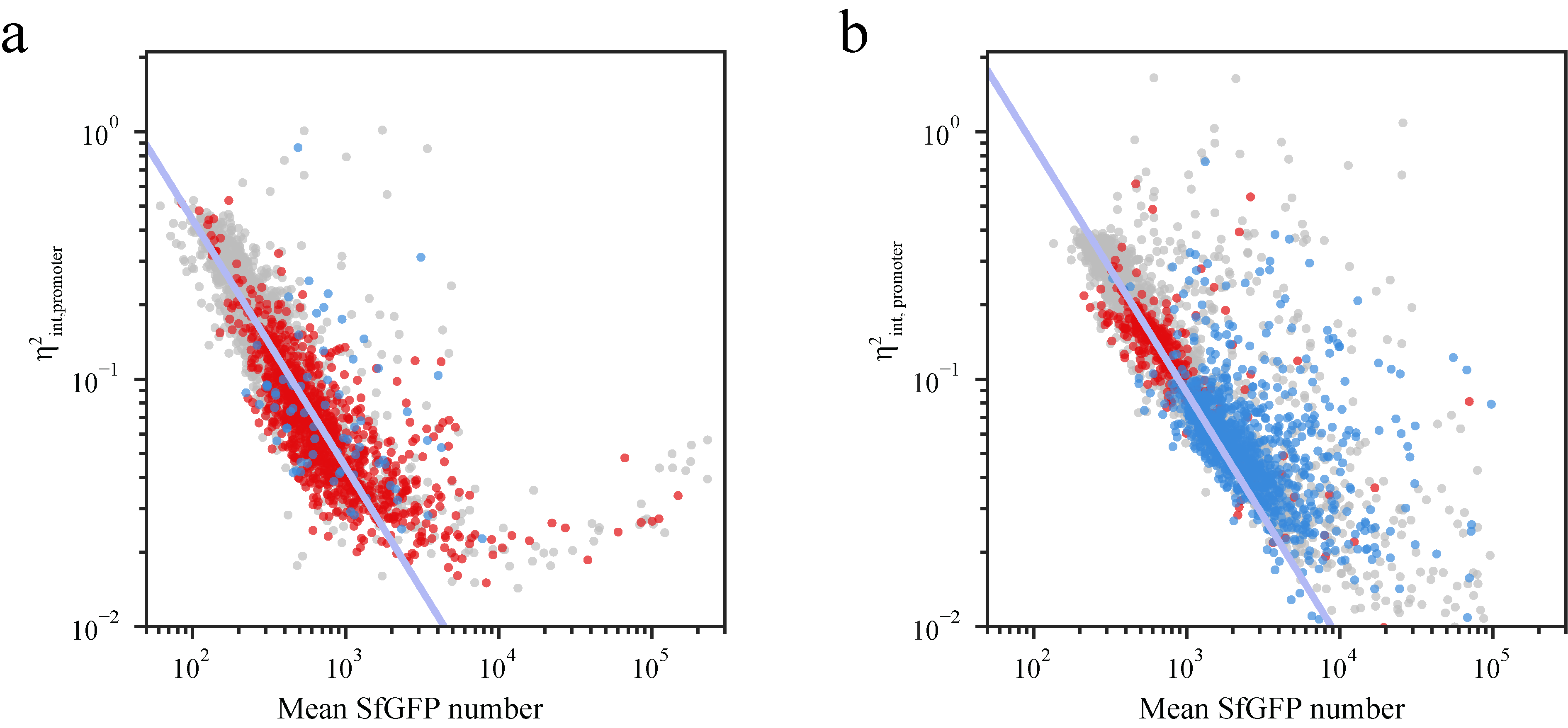

Supplement: FIG S1 [file msystems.00963-22-s0001.tif]

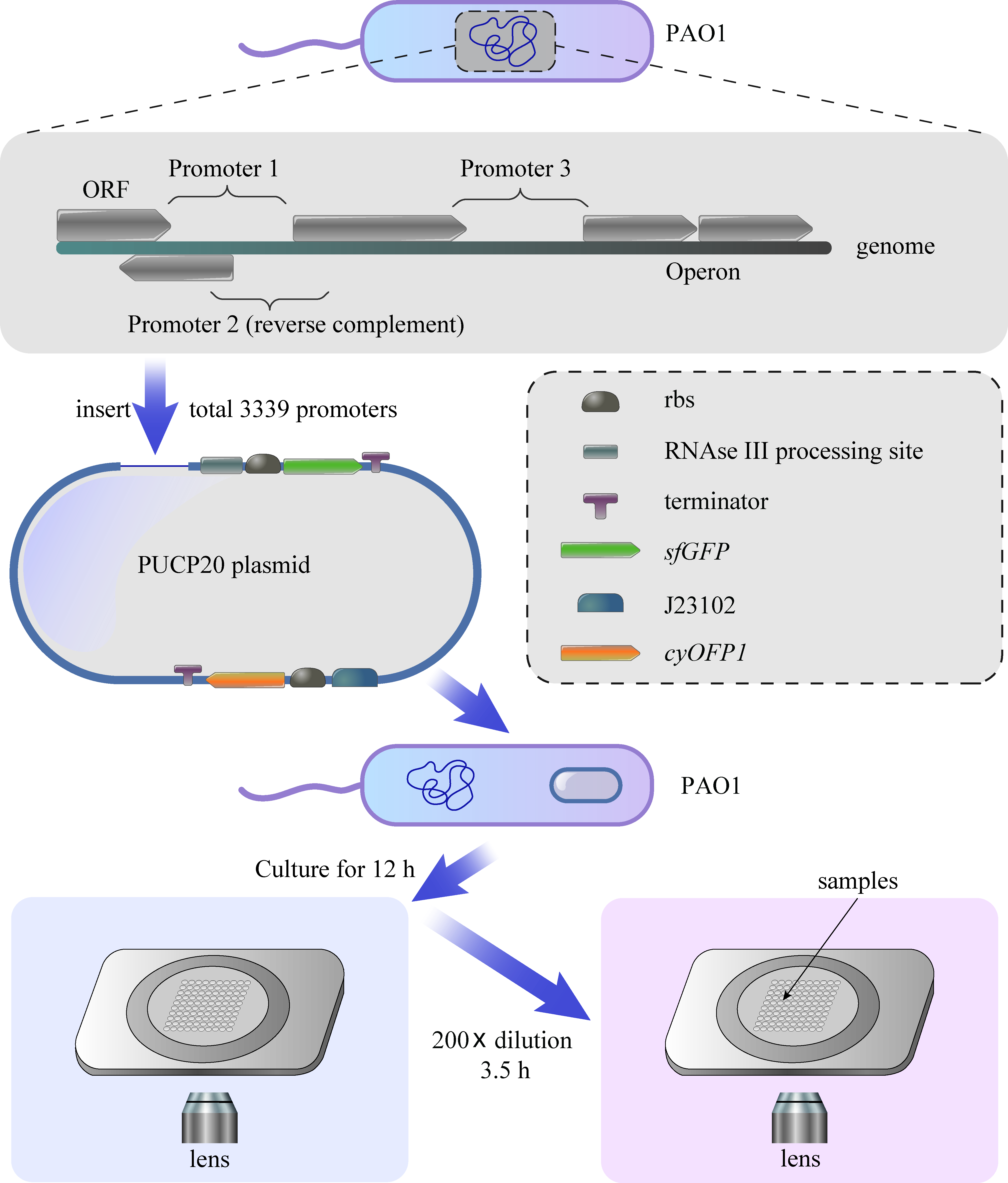

Supplement: FIG S2 [file msystems.00963-22-s0002.tif]

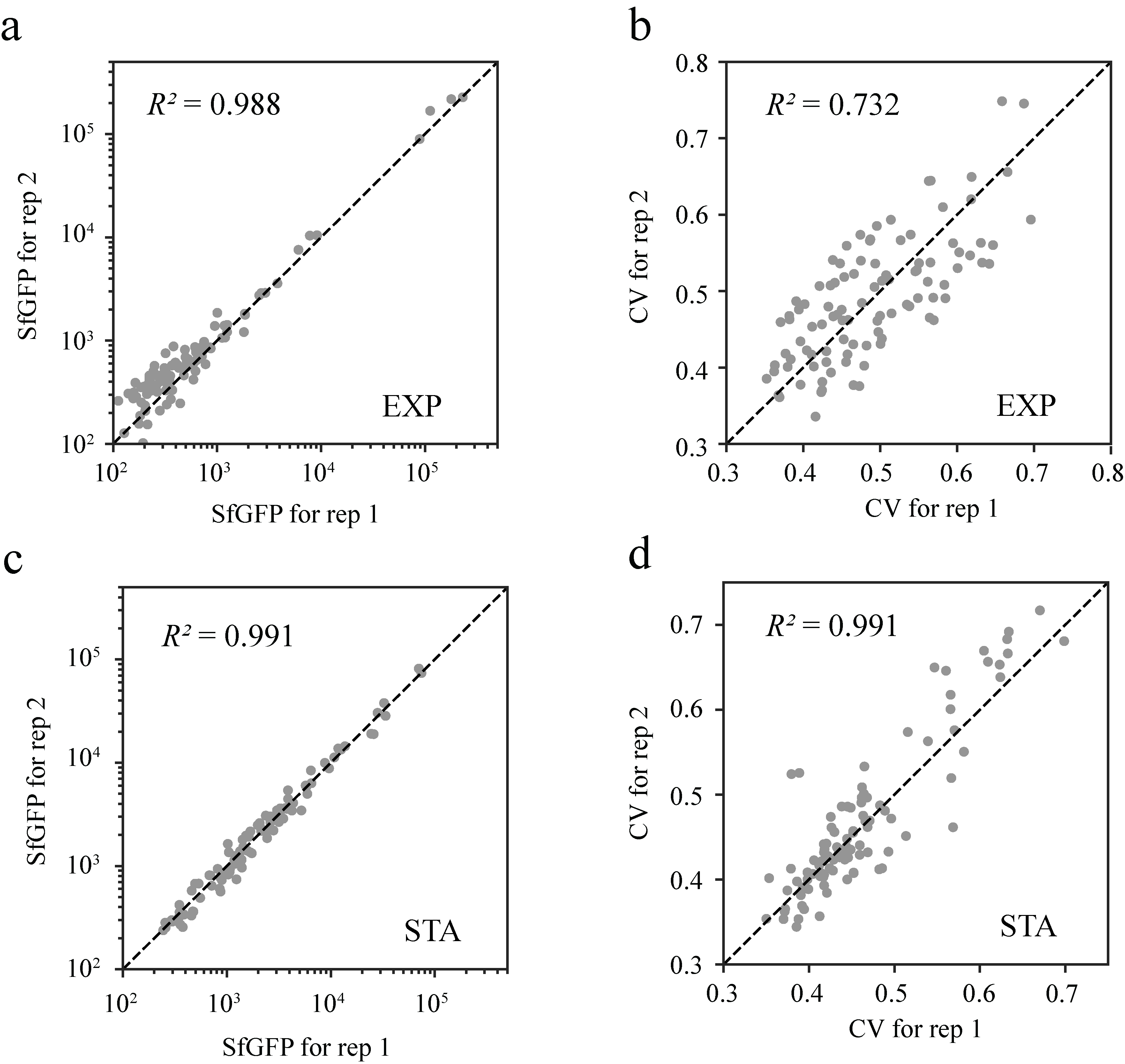

Supplement: FIG S3 [file msystems.00963-22-s0003.tif]

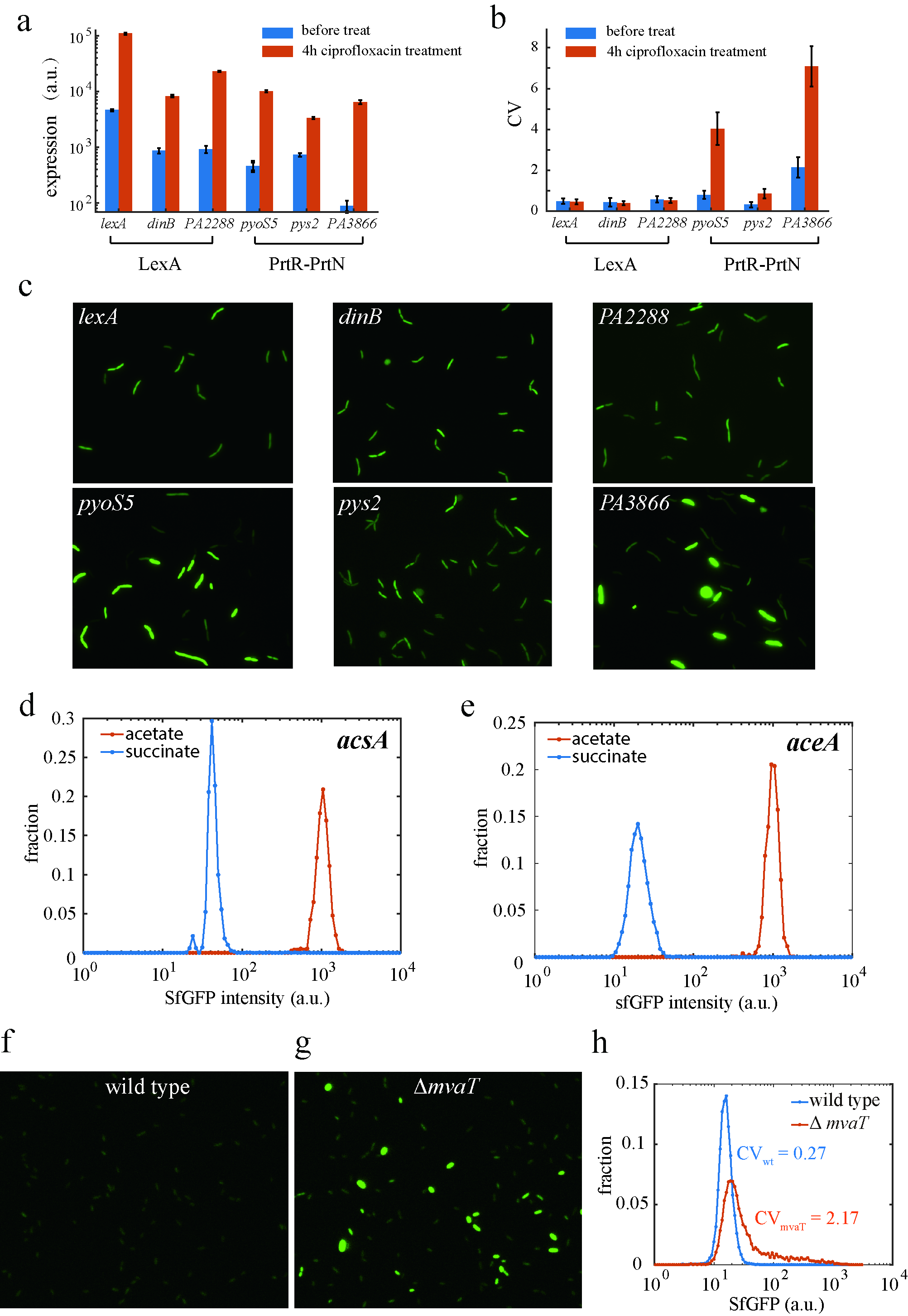

Supplement: FIG S4 [file msystems.00963-22-s0004.tif]

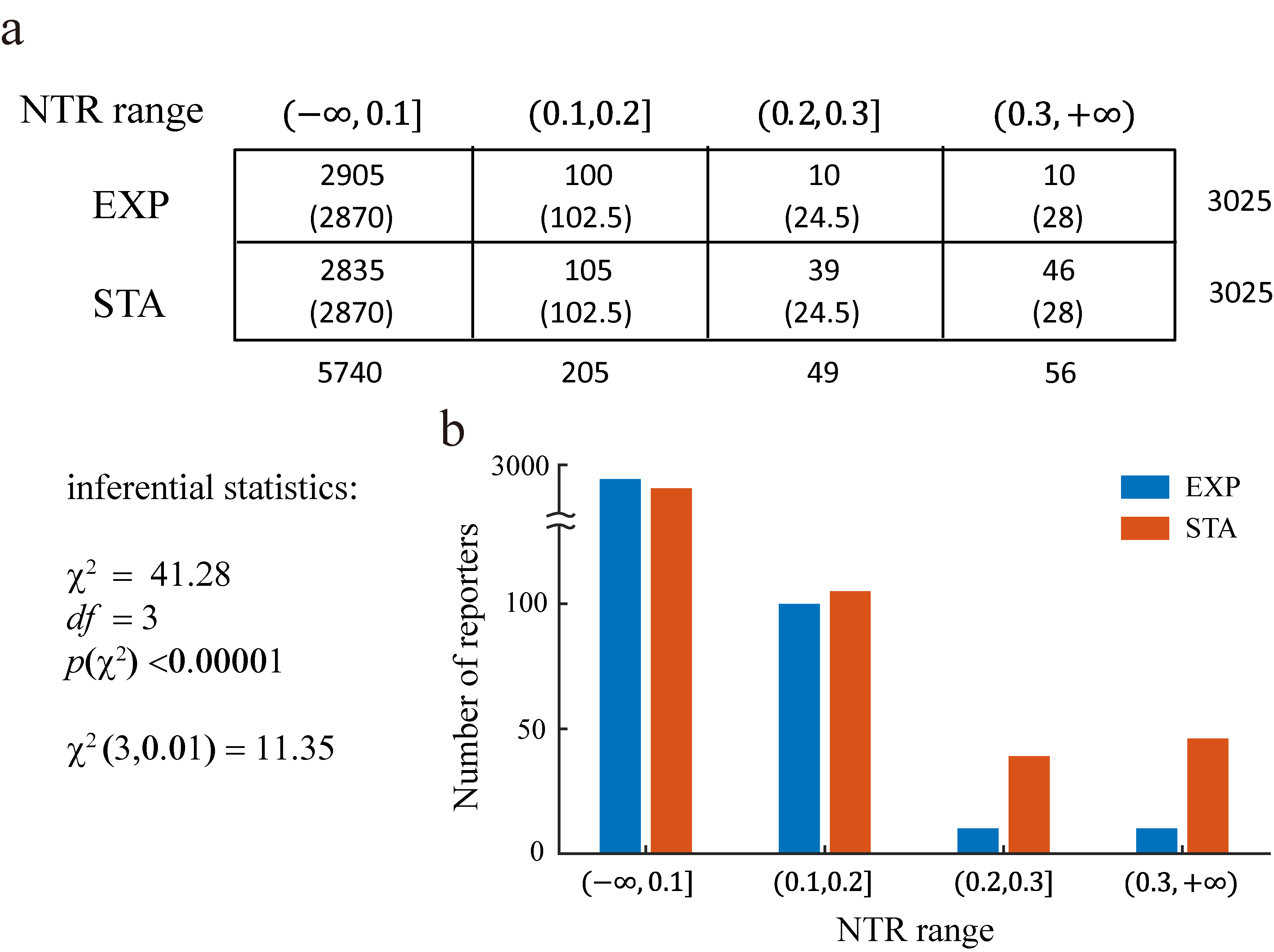

Supplement: FIG S5 [file msystems.00963-22-s0005.tif]

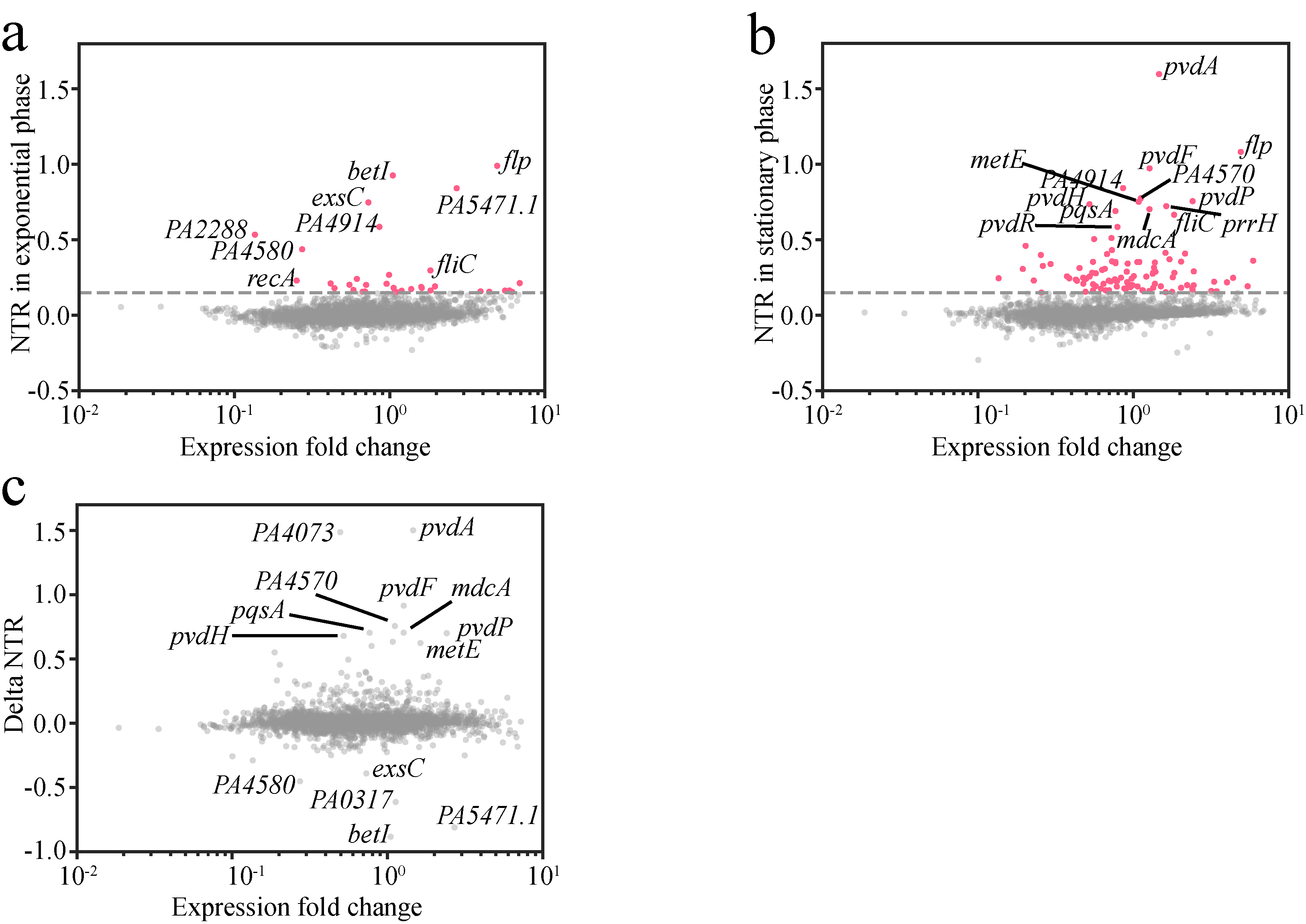

Supplement: FIG S6 [file msystems.00963-22-s0006.tif]

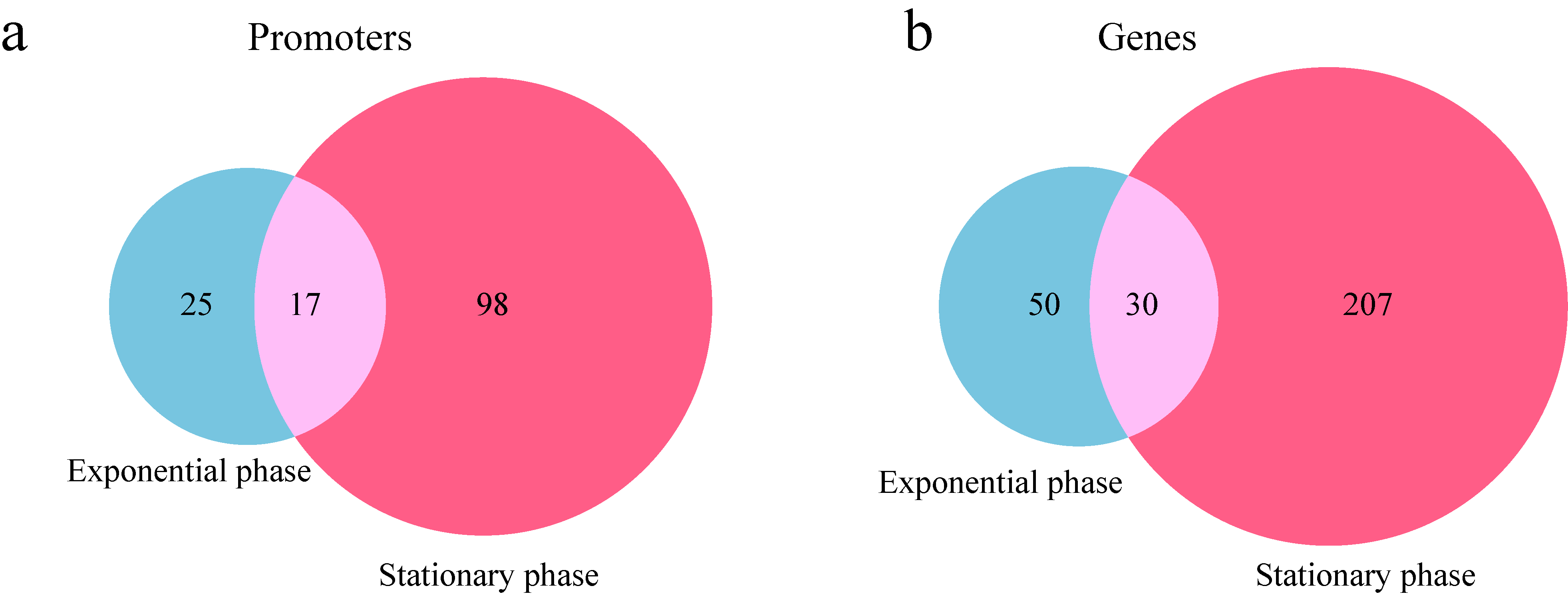

Supplement: FIG S7 [file msystems.00963-22-s0007.tif]

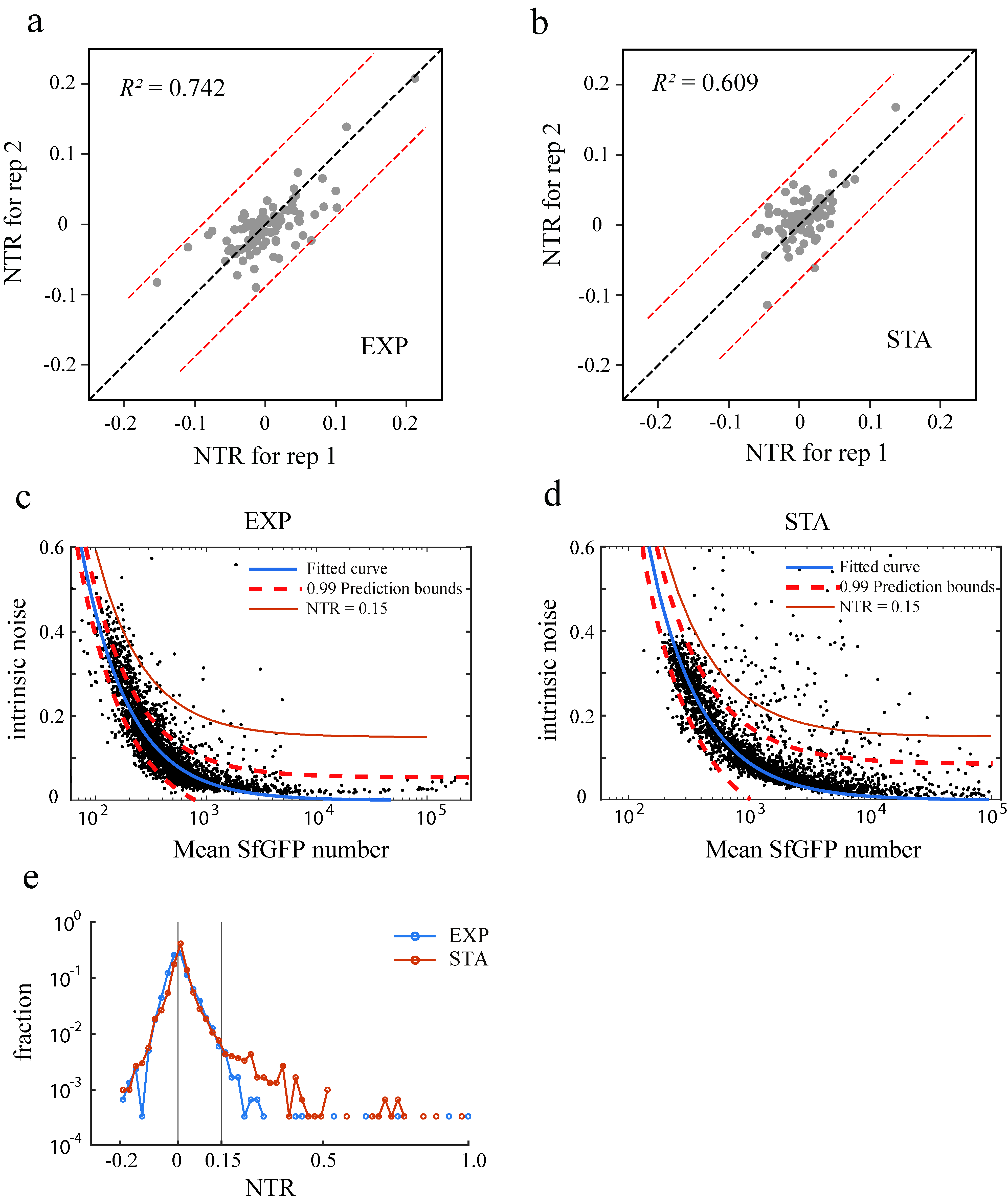

Supplement: FIG S8 [file msystems.00963-22-s0008.tif]
